# Actual anterior–posterior corneal radius ratio in eyes with prior myopic laser vision correction according to axial length

**DOI:** 10.1038/s41598-023-41062-z

**Published:** 2023-08-31

**Authors:** Seung Hee Yoon, Jae Ryong Song, Seung Hyen Lee, Youngsub Eom, Joon Young Hyon, Hyun Sun Jeon

**Affiliations:** 1 Department of Ophthalmology, Seoul National University College of Medicine, Seoul, Republic of Korea; 2 Department of Ophthalmology, Seoul National University College of Medicine, Seoul National University Bundang Hospital, 82, Gumi-Ro 173Beon-Gil, Bundang-Gu, Seongnam-Si, Gyeonggi-Do Republic of Korea; 3 Department of Ophthalmology, Nowon Eulji Medical Center, Eulji University College of Medicine, Seoul, Republic of Korea; 4 Department of Ophthalmology, Korea University College of Medicine, Seoul, Republic of Korea; 5 Department of Ophthalmology, Korea University Ansan Hospital, 123, Jeokgeum-ro, Danwon-gu, Ansan-si, Gyeonggi-do 15355 Republic of Korea; 6 Department of Ophthalmology, Emory University School of Medicine, Atlanta, GA USA

**Keywords:** Eye diseases, Optical materials and structures, Visual system

## Abstract

We retrospectively evaluate the actual anterior–posterior (AP) corneal radius ratio in eyes with previous laser correction for myopia (M-LVC) according to axial length (AL) using biometry data exported from swept-source optical coherence tomography between January 2018 and October 2021 in a tertiary hospital (1018 eyes with a history of M-LVC and 19,841 control eyes). The AP ratio was significantly higher in the LVC group than in the control group. Further, it was significantly positively correlated with AL in the LVC group. We also investigated the impact of the AP ratio, AL and keratometry (K) on the absolute prediction error (APE) in 39 eyes that underwent cataract surgery after M-LVC**.** In linear regression analyses, there were significant correlations between APE and AL/TK, while APE and AP ratio had no correlation. The APE was significantly lower in the Barrett True-K with total keratometry (Barrett True-TK) than in the Haigis-L formula on eyes with AL above 26 mm and K between 38 and 40 D. In conclusion, in eyes with previous M-LVC, AP ratio increases with AL. The Barrett True-K or Barrett True-TK formulas are recommended rather than Haigis-L formula in M-LVC eyes with AL above 26 mm and K between 38 and 40D.

## Introduction

While the development of surgical techniques for myopic laser vision correction (M-LVC) has provided excellent vision without glasses in young people, the improvement of surgical techniques for cataract surgery and advancements in intraocular lenses (IOLs) have also provided excellent vision in middle-aged people. The longer the history of M-LVC, the more patients undergoing cataract surgery after M-LVC^1^. However, it remains a challenge to obtain excellent results after cataract surgery in eyes with a history of M-LVC such as laser-assisted in situ keratomileusis (LASIK), laser epithelial keratomileusis (LASEK), and photorefractive keratectomy (PRK)^2–7^.

The hyperopic error in eyes after previous M-LVC is mainly due to three factors: difficulty in measuring central corneal curvature accurately, prediction of effective lens position, and corneal power^8–12^. Corneal power can be calculated by corneal thickness and anterior and posterior corneal curvature radii^9^. Previously, when anterior corneal curvature radius was measured using a conventional keratometer, posterior corneal curvature data were not available. This limitation resulted in the use of standard refractive indices, mainly the Gullstrand schematic eye of 1.3315 or classical keratometric corneal refractive index of 1.3375^13, 14^, to calculate the total corneal refractive power based on the assumption that the anterior–posterior (AP) corneal curvature radius ratio is constant. However, this assumption does not apply to most previous patients with M-LVC; usually only the anterior corneal curvature becomes flattened, whereas the posterior corneal curvature remains unchanged^15^. As a result, total corneal power is overestimated, leading to the underestimation of IOL power and postoperative hyperopic errors. It is now possible to estimate the posterior corneal curvature radius accurately using Scheimpflug tomography or anterior segment optical coherence tomography (AS-OCT)^16, 17^. Several studies have shown that using these devices can improve the accuracy of estimating total corneal refractive power, leading to minimize prediction errors^18–20^. Previously, some researchers investigated the impact of M-LVC on the keratometric refractive index and evaluated the accuracy of IOL power calculation using the adjusted corneal power based on the AP ratio^21–25^. In line with these studies, first, the present study aimed to investigate the actual AP ratio in M-LVC eyes according to axial length (AL) using raw data exported from IOL Master 700 (Carl Zeiss Meditec, Jena, Germany). Second, we aimed to investigate the impact of the AP ratio, AL, and keratometry (K) on the absolute prediction error (APE) in cataract surgery by comparing the accuracy of the widely used post-LASIK IOL power calculation formulas (Haigis-L and Barrett True-K no history) and Barrett True-K with TK, which utilizes total keratometry (TK) rather than anterior keratometry, in eyes with previous M-LVC.

## Results

### Actual AP ratio in M-LVC eyes according to axial length (AL) and correlations between AP ratio and various biometric parameters

Patient characteristics and ocular biometric parameters of the M-LVC and control groups are shown in Table 1. The M-LVC group consisted of 1018 eyes of 635 patients (209 men, 426 women) and had a mean age of 45.5 ± 11.1 (range 20–81) years. The control group consisted of 19,841 eyes of 10,406 patients (4995 men, 5411 women) and had a mean age of 44.0 ± 26.5 (range 20–98) years. The mean AP ratio was significantly higher in the M-LVC group (1.24 ± 0.05 [range 1.07–1.54]) than in the control group (1.13 ± 0.02 [range 1.04–1.54], p < 0.001, Mann–Whitney U test). The mean AL was significantly longer in the M-LVC group (26.40 ± 1.69 [range 21.59–34.20] mm) than in the control group (24.67 ± 1.77 [range 15.11–36.82] mm, p < 0.001, Mann–Whitney U test). All biometric parameters (anterior corneal radius, posterior corneal radius, CCT, K, and TK), except ACD (p = 0.11), were significantly different between the two groups (p < 0.001, Mann–Whitney U test).

**Table 1:** Patient demographics and ocular biometric parameters of the myopic laser vision correction group, subgroups classified by axial length, and control group.

	M-LVC group					Control group	P-value*
	Total	Group 1 AL < 26.0 mm	Group 2 26.0 mm ≤ AL < 28.0 mm	Group 3 28.0 mm ≤ AL < 30.0 mm	Group 4 30.0 mm ≤ AL		
Eyes	1018	463	406	111	38	19,841	
Age (years)	45.5 ± 11.1	45.5 ± 11.1	44.1 ± 10.5	47.4 ± 12.4	52.5 ± 7.8	44.0 ± 26.5	< 0.001
M:F	209:426					4995:5411	< 0.001
Ant. corneal radius (mm)	8.61 ± 0.49	8.27 ± 0.01	8.83 ± 0.01	9.03 ± 0.04	9.21 ± 0.11	7.72 ± 0.27	< 0.001
Post. corneal radius (mm)	6.90 ± 0.28	6.78 ± 0.01	6.99 ± 0.01	7.05 ± 0.02	6.96 ± 0.07	6.80 ± 0.27	< 0.001
AP ratio^†^	1.24 ± 0.05	1.22 ± 0.04	1.26 ± 0.05	1.28 ± 0.05	1.32 ± 0.07	1.13 ± 0.02	< 0.001
CCT (mm)	0.48 ± 0.04	0.49 ± 0.00	0.47 ± 0.00	0.47 ± 0.00	0.48 ± 0.00	0.54 ± 0.03	< 0.001
AL (mm)	26.40 ± 1.69	25.03 ± 0.03	26.85 ± 0.02	28.73 ± 0.05	31.29 ± 0.17	24.67 ± 1.77	< 0.001
ACD (mm)	3.44 ± 0.31	3.36 ± 0.01	3.50 ± 0.01	3.57 ± 0.03	3.54 ± 0.04	3.39 ± 0.46	0.11
K (D)	39.29 ± 2.23	40.85 ± 0.07	38.25 ± 0.07	37.48 ± 0.20	36.83 ± 0.50	43.74 ± 1.53	< 0.001
TK (D)	38.75 ± 2.40	40.42 ± 0.07	37.64 ± 0.08	36.81 ± 0.22	35.99 ± 0.52	43.73 ± 1.54	< 0.001

Values are presented as mean ± standard deviation.

*Mann–Whitney U test was performed between the M-LVC and control groups.

^†^The Friedman test with Bonferroni post-hoc correction, all p-values < 0.001 between the AP ratio of each subgroup of M-LVC according to AL.

Statistical significance was defined as p < 0.05.

M-LVC, myopic laser vision correction; AP, anterior–posterior; CCT, central corneal thickness; AL, axial length; ACD, anterior chamber depth; K, standard keratometry; TK, total keratometry.

**Figure 1:**
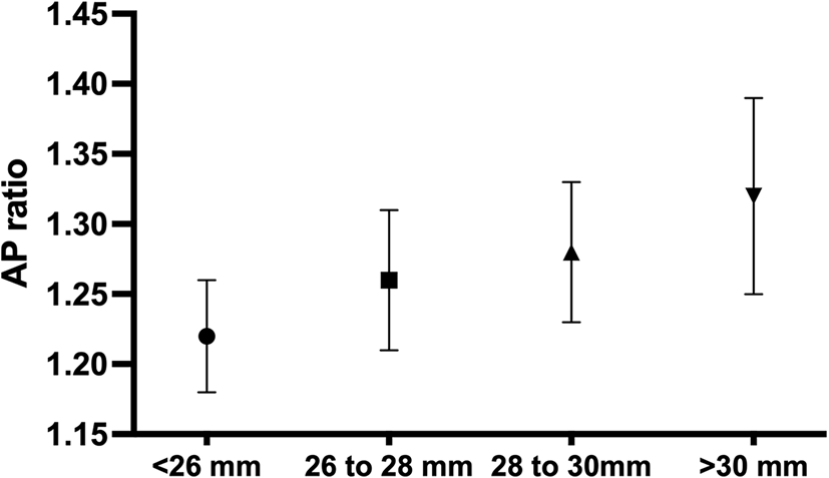
Anterior–posterior corneal radius ratio (AP ratio) of subgroups based on axial length in eyes with myopic laser vision correction. The AP ratios are significantly different among the groups (1.22 ± 0.04, 1.26 ± 0.05, 1.28 ± 0.05, and 1.32 ± 0.07, p < 0.001). In total, 463 eyes with AL < 26.0 mm were included in Group 1, 406 eyes with 26.0 mm $\le$ AL < 28.0 mm in Group 2, 111 eyes with 28.0 mm $\le$ AL < 30.0 mm in Group 3, and 38 eyes with AL > 30.0 mm in Group 4. AL, axial length.

We classified the M-LVC group into four subgroups according to the AL; Group 1 (463 eyes, AL < 26.0 mm), Group 2 (406 eyes, 26.0 mm $\le$ AL < 28.0 mm), Group 3 (111 eyes, 28.0 mm $\le$ AL < 30.0 mm), and Group 4 (38 eyes, AL > 30.0 mm). The AP ratio was significantly different among the groups (1.22 ± 0.04, 1.26 ± 0.05, 1.28 ± 0.05, and 1.32 ± 0.07; p < 0.001, Friedman test with Bonferroni post-hoc correction, Fig. 1).

In the correlation analysis, the AP ratio was significantly correlated with all the other biometric parameters in the control group (p < 0.001, Spearman’s rank correlation coefficient) and with all parameters, except ACD (p = 0.219), in the M-LVC group (p < 0.001, Spearman’s rank correlation coefficient). Considering the correlation coefficients, weak correlations were observed in the control group, whereas the correlations between the AP ratio and other biometric parameters in the M-LVC group were strong, especially in the anterior corneal radius (p < 0.001, r = 0.656), AL (p < 0.001, r = 0.523), K (p < 0.001, r = –0.656), and TK (p < 0.001, r = –0.715).

### Comparison of prediction errors among three IOL power formulas in eyes with previous M-LVC according to axial length and keratometry

**Figure 2:**
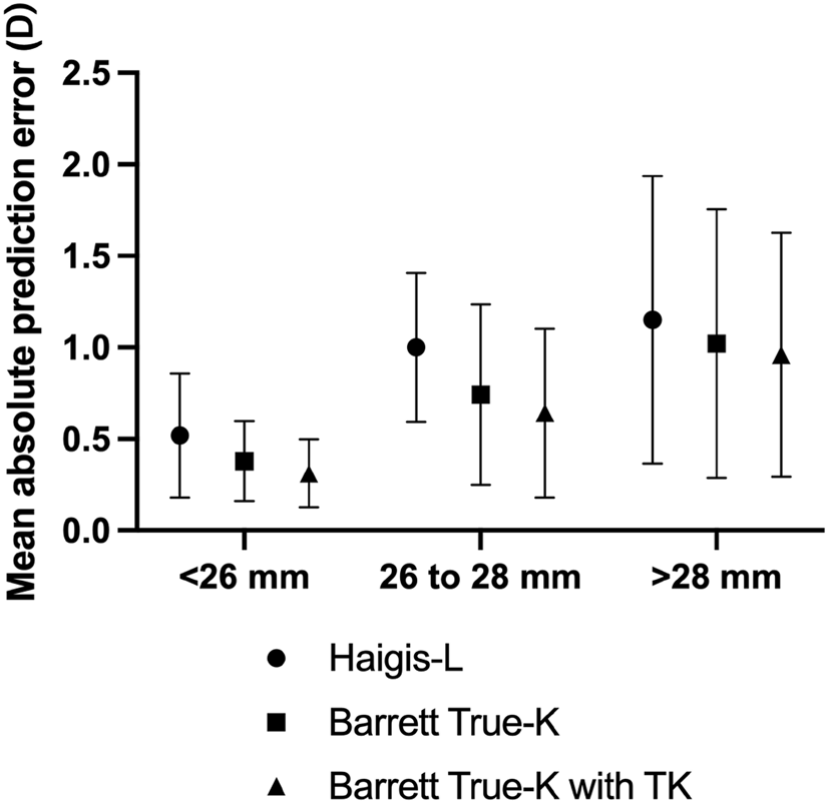
Distribution of mean absolute prediction error calculated with three intraocular lens power formulas across three subgroups according to axial length. Group A, AL < 26.0 mm; Group B, 26.0 mm ≤ AL < 28.0 mm; and Group C, 28.0 mm ≤ AL. AL, axial length; TK, total keratometry.

For analysis of patients with a history of M-LVC who underwent cataract surgery, 39 eyes of 31 patients (11 men, 20 women) were enrolled (Table 2). The mean AL and AP ratio were 27.73 ± 2.39 mm and 1.24 ± 0.07, respectively. We classified the eyes into three subgroups by AL: Group A (AL < 26.0 mm), Group B (26.0 mm $\le$ AL < 28.0 mm), and Group C (28.0 mm $\le$ AL). The mean AP ratios were 1.21 ± 0.05, 1.22 ± 0.02, and 1.28 ± 0.09 in Groups A, B, and C, respectively. Table 3 shows the mean APE (MAE), median APE (MedAE), and percentage of APE within 0.5 and 1.0 D of three IOL power calculation formulas (Haigis-L, Barrett True-K, and Barrett True-TK) for subgroups according to AL. In the case of Group A, no significant difference was found between the MedAEs of each formula. For Groups B and C, Haigis-L had significantly higher MedAE than Barrett True-TK (p = 0.014 and p = 0.04, respectively, Friedman test with Bonferroni post-hoc correction). In Group B, the percentage of APE within 1.0 D was significantly lower in Haigis-L (30.8%) than in Barrett True-K (84.6%) and Barrett True-TK (92.3%; p = 0.048 and p = 0.024, respectively; Cochran Q test with a Bonferroni correction). The distribution of the APE for each subgroup is shown in Fig. 2.

**Table 2:** Patient demographics and ocular biometric parameters on 39 eyes with cataract surgery after myopic laser vision correction according to axial length (Group A–C) and keratometry (Group D–F).

	Total	Group A AL < 26.0 mm	Group B 26.0 mm ≤ AL < 28.0 mm	Group C 28.0 mm ≤ AL	P-value
Eyes	39	10	13	16	
Age (years)	61.46 ± 7.58	61.1 ± 8.71	62.53 ± 6.23	60.81 ± 8.22	0.82
Ant. corneal radius (mm)	8.59 ± 0.54	8.03 ± 0.28	8.57 ± 0.25	8.95 ± 0.54	< 0.001
Post. corneal radius (mm)	6.89 ± 0.29	6.64 ± 0.19	6.98 ± 0.19	6.98 ± 0.33	0.01
AP ratio	1.24 ± 0.07	1.21 ± 0.05	1.22 ± 0.02	1.28 ± 0.09	0.02
CCT (mm)	0.49 ± 0.04	0.52 ± 0.06	0.47 ± 0.03	0.49 ± 0.03	0.07
AL (mm)	27.73 ± 2.39	25.10 ± 0.75	26.94 ± 0.55	30.02 ± 1.78	< 0.001
ACD (mm)	3.35 ± 0.26	3.27 ± 0.36	3.31 ± 0.20	3.44 ± 0.22	0.22
K (D)	39.37 ± 2.47	42.05 ± 1.54	39.39 ± 1.11	37.67 ± 2.28	< 0.001
TK (D)	38.83 ± 2.69	41.67 ± 1.75	38.93 ± 1.17	36.97 ± 2.52	< 0.001

	Total	Group D K < 38.0D	Group E 38.0D ≤ K < 40.0D	Group F 40.0D ≤ K	P-value
Eyes	39	10	15	14	
Age (years)	61.46 ± 7.58	58.60 ± 6.73	64.73 ± 6.78	60.00 ± 8.14	0.09
Ant. corneal radius (mm)	8.59 ± 0.54	9.28 ± 0.46	8.59 ± 0.09	8.09 ± 0.27	< 0.001
Post. corneal radius (mm)	6.89 ± 0.29	7.16 ± 0.32	6.93 ± 0.17	6.66 ± 0.18	< 0.001
AP ratio	1.24 ± 0.07	1.29 ± 0.11	1.24 ± 0.03	1.21 ± 0.04	0.015
CCT (mm)	0.49 ± 0.04	0.50 ± 0.03	0.47 ± 0.03	0.51 ± 0.05	0.071
AL (mm)	27.73 ± 2.39	30.75 ± 1.82	27.71 ± 1.07	25.59 ± 1.09	< 0.001
ACD (mm)	3.35 ± 0.26	3.39 ± 0.19	3.39 ± 0.25	3.28 ± 0.31	0.511
K (D)	39.37 ± 2.47	36.21 ± 1.65	39.27 ± 0.44	41.72 ± 1.46	< 0.001
TK (D)	38.83 ± 2.69	35.46 ± 2.02	38.75 ± 0.48	41.32 ± 1.63	< 0.001

Values are presented as mean ± standard deviation. AP, anterior–posterior; CCT, central corneal thickness; AL, axial length; ACD, anterior chamber depth; K, standard keratometry; TK, total keratometry.

Statistical significance was defined as p < 0.05.

**Table 3:** Median and mean absolute prediction errors and percentage of prediction error within ± 0.5 D and ± 1 D of three intraocular lens power calculation formulas in subgroups classified by axial length (Group A-C) and keratometry (Group D-F).

	Haigis-L	Barrett True-K	Barrett True-TK	P-value
Group A (AL < 26.0 mm, n = 10)				
MedAE (MAE)	0.62 (0.51 ± 0.33)	0.32 (0.37 ± 0.21)	0.25 (0.31 ± 0.18)	0.14
± 0.5 D, (%)	50	80	70	0.09
± 1 D, (%)	100	100	100	> 0.99
Group B (26.0 mm ≤ AL < 28.0 mm, n = 13)				
MedAE (MAE)	1.06 (1.00 ± 0.40)	0.73 (0.74 ± 0.49)	0.57 (0.64 ± 0.46)	0.017*
± 0.5 D, (%)	7.7	23.1	30.8	0.17
± 1 D, (%)	30.8	84.6	92.3	**0.001**^**†**^
Group C (28.0 mm ≤ AL, n = 16)				
MedAE (MAE)	1.09 (1.15 ± 0.78)	0.99 (1.02 ± 0.73)	0.87 (0.96 ± 0.66)	**0.04**^**‡**^
± 0.5 D, (%)	31.3	31.3	31.3	> 0.99
± 1 D, (%)	43.8	50	50	0.77
Group D (K average < 38.0D, n = 10)				
MedAE (MAE)	1.67 (1.66 ± 0.60)	1.44 (1.35 ± 0.63)	1.30 (1.26 ± 0.56)	0.05
± 0.5 D, (%)	10	0	0	0.36
± 1 D, (%)	10	30	30	0.26
Group E (38.0D ≤ K average < 40.0D, n = 15)				
MedAE (MAE)	0.90 (0.78 ± 0.40)	0.72 (0.60 ± 0.43)	0.43 (0.51 ± 0.38)	**0.009**^**§**^
± 0.5 D, (%)	26.7	33.3	53.3	**0.03**^**¶**^
± 1 D, (%)	53.3	86.7	93.3	0.02**
Group F (40.0D ≤ K average, n = 14)				
MedAE (MAE)	0.76 (0.58 ± 0.38)	0.38 (0.51 ± 0.47)	0.30 (0.45 ± 0.45)	0.23
± 0.5 D, (%)	42.9	71.4	57.1	0.05
± 1 D, (%)	85.7	92.9	92.9	0.36

Values are presented as mean ± standard deviation.

* Haigis-L and Barrett True-TK: p = 0.014 using the Friedman test with Bonferroni post-hoc correction.

^†^Haigis-L and Barrett True-K: p = 0.048, Haigis-L and Barrett True-TK: p = 0.024 using the Cochran Q test with a Bonferroni correction.

^‡^Haigis-L and Barrett True-TK: p = 0.04 using the Friedman test with Bonferroni post-hoc correction.

^§^ Haigis-L and Barrett True-K: p = 0.02 using the Friedman test with Bonferroni post-hoc correction.

Haigis-L and Barrett True-TK: p = 0.02 using the Friedman test with Bonferroni post-hoc correction.

^¶^ Haigis-L and Barrett True-TK: p = 0.046 using the Cochran Q test with a Bonferroni correction.

** Haigis-L and Barrett True-TK: p = 0.034 using the Cochran Q test with a Bonferroni correction.

Statistical significance was defined as p < 0.05.

AL, axial length; MAE, mean absolute error; MedAE, median absolute error; TK, total keratometry.

Significant values are in [bold].

For additional subgroup analysis, we classified the eyes into three subgroups by keratometry: Group D (K < 38.0D), Group E (38.0D $\le$ K < 40.0D), and Group F (40.0D $\le$ K; Table 2). The mean AP ratios were 1.29 ± 0.11, 1.24 ± 0.03, and 1.21 ± 0.04 in Groups D, E, and F, respectively. For Group D and F, no significant difference was found among the MedAEs of each formula; MedAEs were high in Group D and low in Group F. For Groups E, Haigis-L had significantly higher MedAE than Barrett True-K and Barrett True-TK (p = 0.02 for both case, Friedman test with Bonferroni post-hoc correction). In Group E, the percentage of APE within 0.5 D was significantly lower in Haigis-L (26.7%) than in Barrett True-TK (53.3%; p = 0.046, Cochran Q test with a Bonferroni correction). Also, the percentage of APE within 1.0 D was significantly lower in Haigis-L (53.3%) than in Barrett True-TK (93.3%; p = 0.034, Cochran Q test with a Bonferroni correction).

In linear regression analyses of the relationship between the AP ratio and APE of three IOL power formulas, no correlation between the AP ratio and APE was statistically significant (p = 0.06, 0.94, and 0.11 for Haigis-L, Barrett True-K, and Barrett True-TK, respectively). The APEs of Haigis-L and Barrett True-TK tended to increase as the AP ratio increased, whereas the APE of Barrett True-K tended to decrease slightly as the AP ratio increased. Meanwhile, linear regression analyses of the relationship between AL and APE of three IOL power formulas revealed statistically significant correlations in each formula (p < 0.001, p = 0.001, p < 0.001 for Haigis-L, Barrett True-K, and Barrett True-TK, respectively). Additionally, linear regression analyses of the relationship between TK and APE of three IOL power formulas also showed statistically significant correlations in each formula (p = 0.003, p = 0.026, p = 0.037 for Haigis-L, Barrett True-K, and Barrett True-TK, respectively).

## Discussion

This study investigated the actual AP ratio in both M-LVC and naïve eyes using a large amount of raw data exported from the IOL Master 700. We confirmed that the mean AP ratios of M-LVC and control naïve eyes were 1.24 ± 0.05 and 1.13 ± 0.02, respectively, whereas the Gullstrand schematic eye model suggests an AP ratio of 1.13. Previous studies reported AP ratios in normal corneas ranging from 1.19 to 1.23^13, 26–31^. This discrepancy may be due to differences in the principles of the devices used for measuring corneal curvatures or due to racial differences^31^. However, results from our data from large number of 19,841 naïve eyes suggest that the AP ratio of the normal cornea converges to that of the Gullstrand schematic eye model, implying that the AP ratio may not affect postoperative error in normal corneas. Conversely, as expected, the mean AP ratio in the M-LVC eyes was significantly higher than that in normal eyes, which implies that the AP ratio may affect prediction errors and should be considered when selecting IOL power. To the best of our knowledge, no studies have reported the actual AP ratio according to AL in eyes with M-LVC. We focused on the finding that AL had a strong correlation with the AP ratio in the M-LVC group (p < 0.001, r = 0.523) and found that the AP ratio was significantly higher in subgroups with long AL. From our results, we can explain the higher prediction errors in M-LVC eyes, especially in eyes with long AL. Our results suggest that greater attention should be paid to the AP ratio in cases of high myopia with extremely long AL. It can be expected that the formula using TK, which reflects AP ratio will be more accurate in those eyes.

The results of the correlations between the AP ratio and the other biometric parameters, as expected, revealed that all parameters, except for ACD, were significantly correlated with AP ratio in the M-LVC, which is consistent with the results of previous studies on LVC^28, 32, 33^. Correlation analyses, which have shown strong correlations between the AP ratio and anterior corneal radius, AL, K, and TK, suggest that the AP ratio can be an important factor in eyes with a history of M-LVC.

We compared the accuracy of the widely used post-LASIK IOL power calculation formulas (Haigis-L and Barrett True-K no history) and Barrett True-TK, which utilizes TK value from IOL Maser700. Recent studies showed that methods using no previous data, including the Haigis-L and Barrett True-K provided better results than methods using pre-LVC values^34–36^. The Haigis-L formula is a modified form of the regular Haigis formula utilizing a regression-based algorithm, which generates a corrected central corneal power based on the corneal radius, thereby relatively accurately predicting the myopic LASIK eyes for extremely long eyes^37^. The Barrett True-K formula is based on the Barrett Universal II formula and calculates the modified K value and applies the double K method^38^. We hypothesized that for the Haigis-L and Barrett True-K formulas, which do not consider posterior corneal radii curvature, the AP ratio and APE may have statistically significant correlation on simple linear regression analysis. However, our study results did not support our hypothesis, since none of the APE showed a significant correlation with the AP ratio, while AL and TK both had significant correlation with APE of each formula, each with positive and negative beta coefficient in linear regression analysis. These results can be interpreted that eyes with long AL or low TK are likely to have a large amount of refractive correction, and thus the AP ratio has increased. In the contrary, Huh et al.^22^ which also used AP ratio found positive coefficients between the two values (AP ratio and APE). This discrepancy may be caused by the difference in patients characteristics and the limited number of eyes, and further studies including more patients are needed.

We also tried to compare the accuracy of the Barrett True-TK reflecting the actual AP ratio with widely used Haigis-L and Barrett True-K no history methods. In 2022, Fang et al.^39^ found that Haigis-L was relatively accurate in predicting extreme long axis (> 29.0 mm) eyes after M-LVC but less accurate for eyes with extremely flat corneas (< 35 D). In our study, the percentage of APE within 1.0 D was significantly lower in Haigis-L than in Barrett True-K and Barrett True-TK in eyes with 26.0 mm $\le$ AL < 28.0 mm, which is consistent with the previous results^38, 40–42^. Although there was no statistical significance, the Barrett True-TK showed higher prediction accuracy than Barrett True-K, which need to be confirmed in further studies. In addition, the percentage of APE within 0.5 and 1.0 D was significantly lower in Haigis-L than in Barrett True-TK in eyes with 38.0D $\le$ K < 40.0 D. Also, results showed that all three formulas can more accurately predict the IOL power in eyes with 40.0D $\le$ K or AL < 26.0 mm, while all three formulas were less accurate for eyes with extremely long (AL > 28.0 mm) or flat (TK < 38.0 D). This corresponds to the results that IOL power calculation formulas can more accurately predict the IOL power in eyes with AL < 26.0 mm, since longer AL typically induces severe myopia, which leads to flat K after M-LVC.

Similarly, Huh et al.^22^ evaluated the prediction accuracy of IOL calculation using adjusted corneal power according to AP ratio in Haigis formula (Haigis-E) in M-LVC eyes. They also applied Wang-Koch adjustment in eyes longer than 25.0 mm. They reported Haigis-E provided a more accurate estimate of refractive outcomes than the Shammas, Haigis-L, and the Barrett True-K no-history methods. Subgroup analyses in their study also revealed that the prediction accuracy was higher in Barrett True-K than in Haigis-L in eyes longer than 26.0 mm, although it was the highest in Haigis-E. Based on their and our results, AP ratio, which also affected by AL may be considered in post-LASIK IOL calculation, and the Barrett True-K or Barrett True-TK formulas are recommended rather than Haigis-L formula for post-LASIK eyes longer than 26.0 mm.

Although this study excluded patients with other corneal and retinal diseases, inclusion of such patients could significantly change the AP ratio, making it difficult to calculate the IOL power. For example, in patients with advanced keratoconus, postoperative hyperopic shift is caused by overestimation of keratometric value^43, 44^. Tamaoki et al.^44^ reported that four patients with posterior keratoconus who underwent cataract surgery showed a prediction error of 0.975 when partial coherence interferometry-measured K value was applied, whereas when total corneal refractive power using AS-OCT was applied, the error was 0.385. Therefore, additional optimization or modification is required for IOL power calculation in situations related to variations in the AP ratio.

This study has some limitations. First, small number of eyes with cataract surgery after M-LVC were investigated. However, since our study included M-LVC eyes with wide range of AL, we can confirm the influence of various Als on each formula. Second, the correction amount of LVC was not available, which may have affected the values of the biometric parameters. However, considering that there was a difference in the AP ratio depending on the AL, it could be replaced by AL. Further prospective studies including large number of eyes with histories of preoperative refractive errors are required to precisely investigate the correlation between the AP ratio and IOL power calculation prediction errors. Third, the M-LVC group includes eyes that have underwent LASIK, LASEK, and PRK procedures and the impact of the surgical methods on the AP ratio was not taken into consideration. Further research is needed to investigate the impact of surgical methods on the AP ratio. Fourth, as a retrospective analysis, not all participants were evaluated for dry eye including tear break-up time, which could potentially influence ophthalmic biometry. However, we believe that the impact of dry eye, which could influence the ophthalmic biometric results, is minimized as we conduct preoperative evaluations after treating dry eye conditions in patients undergoing cataract surgery.

In conclusion, the AP ratio in eyes with previous M-LVC was significantly higher than that of naïve eyes and it has positive correlation with AL, thus IOL calculation formula which reflecting TK will be more accurate especially in eyes with long AL. Based on our study, the Barrett True-K or Barrett True-TK formulas are recommended rather than Haigis-L formula in M-LVC eyes with AL above 26.0 mm or K between 38.0 and 40.0 D for better refractive outcomes.

## Methods

### Study population

For the first study aim, we included eyes from the raw data exported from a swept-source optical coherence tomography system device (IOLMaster 700), which were obtained between January 2018 and October 2021 at a tertiary hospital in South Korea, Seoul National University Bundang Hospital. We excluded eyes with a lens status of pseudophakia, quality index of “fail” or “warning” in any of the biometric parameters in IOLMaster 700 measurements, and vitreous status of silicon oil. In total, 20,859 eyes of 11,041 patients were included in the measurement. Eyes were then divided into two groups: the M-LVC group, which underwent corneal refractive surgery, including LASIK, LASEK, and PRK, and the control group with no history of M-LVC. The M-LVC and control groups included 1018 eyes of 635 patients and 19,841 eyes of 10,406 patients, respectively.

For the second aim, a retrospective chart review was performed on patients who underwent phacoemulsification with IOL implantation using ZCB00 (Johnson & Johnson Vision Care, Inc., Santa Ana, CA, USA) after M-LVC (LASIK, LASEK, PRK) and underwent measurements using IOLMaster 700 between January 2018 and October 2021 at Seoul National University Bundang Hospital. We excluded eyes without posterior corneal radius data, as it was necessary to evaluate results using the AP ratio, and the presence of other corneal diseases could affect keratometry. We also excluded patients with marked decentration by confirming topography. In total, 39 eyes from 31 patients were included in the analysis. The study protocol adhered to the tenets of the Declaration of Helsinki and was approved by the Institutional Review Board (IRB) (IRB: B-2201-730-002) of Seoul National University Bundang Hospital. Informed consent was waived owing to the retrospective nature of this study by the IRB of Seoul National University Bundang Hospital (IRB: B-2201-730-002).

### Actual anterior–posterior (AP) ratio in myopic laser vision correction (M-LVC) eyes according to axial length with raw data from IOLMaster 700

Among the exported raw data from IOLMaster 700, anterior and posterior corneal radii, central corneal thickness (CCT), AL, anterior chamber depth (ACD), K, and TK were reviewed for analysis. AP ratio was defined as the anterior corneal radius divided by the posterior corneal radius. We classified eyes with M-LVC into four subgroups according to AL to investigate the effect of AL on the AP ratio. In total, 463 eyes with AL < 26.0 mm were included in Group 1, 406 eyes with 26.0 mm $\le$ AL < 28.0 mm in Group 2, 111 eyes with 28.0 mm $\le$ AL < 30.0 mm in Group 3, and 38 eyes with AL > 30.0 mm in Group 4. Correlation analyses between the AP ratio and age, anterior corneal radius, posterior corneal radius, CCT, AL, ACD, K, and TK were performed.

### Comparison of absolute prediction errors between three intraocular lens (IOL) power formulas in eyes with previous M-LVC according to axial length and keratometry

The prediction error was defined as the postoperative spherical equivalent 1 month after cataract surgery subtracted from the refractive outcome predicted from each formula. We used the APE, which is the absolute value of the prediction error in the analyses. The APEs were compared by three IOL power calculation formulas: Haigis-L, Barrett True-K no history (referred to as “Barrett True-K”), and Barrett True-K with TK (Barrett True-TK). Barrett True-TK formula was defined as a modification of the Barrett True-K formula, additionally calculated by the posterior corneal radius, utilizing the formula provided on the Asia–Pacific Association of Cataract and Refractive Surgeons website. The IOL constants used were A0 of –1.302, A1 of + 0.210, and A2 of + 0.251 for the Haigis L formula and lens factor of + 2.09 and design factor of + 4.0 for Barrett True-K and Barrett True-TK formulas, respectively. The percentages of eyes with a refractive prediction error within ± 0.50 D and ± 1.00 D among the three formulas were compared according to AL subgroups; 10 eyes with AL < 26.0 mm were included in Group A, 13 eyes with 26.0 mm $\le$ AL < 28.0 mm in Group B, and 16 eyes with 28.0 mm $\le$ AL in Group C. The comparison was also performed according to keratometry subgroups; 10 eyes with K < 38.D were included in Group D, 15 eyes with 38.0D $\le$ K < 40.0D in Group E, and 14 eyes with 40.0D $\le$ K in Group F.

### Statistical analyses

SPSS Statistics software (version 23; IBM Corporation, Armonk, NY, USA) was used to perform statistical analyses. The Mann–Whitney U test was performed to compare the biometric parameters between the M-LVC and control groups. The association between the AP ratio and other biometric parameters was analyzed using Spearman’s rank correlation coefficient. The Friedman test with Bonferroni correction was conducted to compare the refractive prediction error between each formula. In addition, the Cochran Q test with a Bonferroni correction was used to compare the percentages of eyes with a refractive prediction error within ± 0.5 D and ± 1.0 D among the formulas. Linear regression analysis was performed to investigate the correlation between APE of each formula and AP ratio, AL, and K. Statistical significance was defined as p < 0.05.

## Data Availability

The datasets generated during and/or analysed during the current study are available from the corresponding author on reasonable request.
